# Learning a Set of Interrelated Tasks by Using a Succession of Motor Policies for a Socially Guided Intrinsically Motivated Learner

**DOI:** 10.3389/fnbot.2018.00087

**Published:** 2019-01-08

**Authors:** Nicolas Duminy, Sao Mai Nguyen, Dominique Duhaut

**Affiliations:** ^1^Département Mathématiques Informatique Statistique, Université Bretagne-Sud, CNRS, Lab-STICC, Lorient, France; ^2^IMT Atlantique, Lab-STICC, Université Bretagne Loire (UBL), Nantes, France

**Keywords:** intrinsic motivation, goal-babbling, multi-task learning, interactive learning, active learning, active imitation learning, hierarchical learning, procedures

## Abstract

We aim at a robot capable to learn sequences of actions to achieve a field of complex tasks. In this paper, we are considering the learning of a set of interrelated complex tasks hierarchically organized. To learn this high-dimensional mapping between a continuous high-dimensional space of tasks and an infinite dimensional space of unbounded sequences of actions, we introduce a new framework called “procedures”, which enables the autonomous discovery of how to combine previously learned skills in order to learn increasingly complex combinations of motor policies. We propose an active learning algorithmic architecture, capable of organizing its learning process in order to achieve a field of complex tasks by learning sequences of primitive motor policies. Based on heuristics of active imitation learning, goal-babbling and strategic learning using intrinsic motivation, our algorithmic architecture leverages our procedures framework to actively decide during its learning process which outcome to focus on and which exploration strategy to apply. We show on a simulated environment that our new architecture is capable of tackling the learning of complex motor policies by adapting the complexity of its policies to the task at hand. We also show that our “procedures” enable the learning agent to discover the task hierarchy and exploit his experience of previously learned skills to learn new complex tasks.

## 1. Introduction

Recently, efforts in the robotic industry and academic field have been made for integrating robots in previously human only environments. In such a context, the ability for service robots to continuously learn new tasks, autonomously or guided by their human counterparts, has become necessary. They would be needed to carry out multiple tasks, especially in open environments, which is still an ongoing challenge in robotic learning. The range of tasks those robots need to learn can be wide and even change after the deployment of the robot. These tasks can also require the execution of complex policies, such as sequences of primitive policies.

Learning to associate a potentially unbounded sequence of policies to a set of infinite tasks is a challenging problem for multi-task learning, because of the high-dimensionality of the policy and state spaces, of multi-task learning, and of the unbounded, continuous and loosely specified environments.

To address these challenges, we examine methods for robots to learn motor policy sequences, methods for multi-task learning, as well as heuristics for learning in high-dimensional mappings such as active learning based on intrinsic motivation, social guidance and strategic learning.

### 1.1. Learning Motor Policy Sequences

In this article, we tackle the learning of complex policies to complete high-level tasks. More concretely, in this study, we define the policies as a sequence of primitive policies. As we wish to get rid of any a priori on the maximum complexity of the policy needed to complete any task, the sequence of primitive policies can be unbounded. The learning agent thus learns to associate to any outcome or effect on the world, an a priori unbounded sequence of primitive policies. We review in this paragraph works in compositionally of primitives from the robot learning perspective.

A first approach to learning motor policies is to use via-points such as in Stulp and Schaal (2011), Reinhart (2017) or parameterized skills such as in da Silva et al. (2012). The number of via-points or parameters is a way to define the level of complexity of the policies, but these works use a fixed and finite number of via-points. A small number of via-points can limit the complexity of the policies available to the learning agent, while a high number can increase the number of parameters to be learned. Another approach is chain primitive actions into sequences of policies. However, this would increase the difficulty for the learner to tackle simpler tasks which would be reachable using less complex policies. Enabling the learner to decide autonomously the complexity of the policy necessary to solve a task would allow the approach to be adaptive, and suitable to a greater number of problems.

Options Sutton et al. (1999) and (Machado et al., 2017) introduced in the reinforcement learning framework (Sutton and Barto, 1998) offer temporally abstract actions to the learner. These options represent a temporal abstraction of policies as explained in Sutton (2006). Chains of options have been proposed as extensions in order to reach a given target event. Learning simple skills and planning sequences of policies instead of learning a sequence directly has been shown to simplify the learning problem in Konidaris and Barto (2009). They are a way to represent policy probability density in a goal-oriented way. However, each option is built to reach one particular task and they have only been tested for discrete tasks and actions, in which a bounded number of options were used. We would like to reuse this idea of temporal abstraction and goal-oriented representation to create unbounded policy sequences.

### 1.2. Multi-Task Learning by a Hierarchical Representation

Indeed, an essential component of autonomous, flexible and adaptive robots will be to exploit temporal abstractions, i.e., to treat complex tasks of extended duration, that is to treat complex tasks of extended duration (e.g., making a drawing) not as a single skill, but rather as a sequential combination of skills (e.g., grasping the pen, moving the pen to the initial position of the drawing, etc.) Such task decompositions drastically reduce the search space for planning and control, and are fundamental to making complex tasks amenable to learning. This idea can be traced back to the hypothesis posed in Elman (1993) that the learning needs to be progressive and develop, *starting small*. It has been renamed as *curriculum learning* in Bengio et al. (2009), as formalized in terms of order of the training dataset: the examples should not randomly presented but organized in a meaningful order which illustrates gradually more concepts, and gradually more complex ones. For multi-task learning in the reinforcement framework, it has been studied as *hierarchical reinforcement learning* as introduced in Barto and Mahadevan (2003), relying on task decomposition or task hierarchy.

Indeed, the relationships between tasks in task hierarchy in Forestier and Oudeyer (2016); Reinhart (2017) have been successfully exploited for learning tool use or learning inverse models for parameterized motion primitives, allowing the robot to reuse previously learned tasks to build more complex ones. As opposed to classical methods enabling robots to learn tool-use, as (Brown and Sammut, 2012) or (Schillaci et al., 2012), which consider tools as objects with affordances to learn using a symbolic representation, (Forestier and Oudeyer, 2016) does not necessitate this formalism and learns tool-use using simply parameterized skills, leveraging on a pre-defined task hierarchy. Barto et al. (2013) showed that building complex actions made of lower-level actions according to the task hierarchy can bootstrap exploration by reaching interesting outcomes more rapidly. Temporal abstraction has also proven to enhance the learning efficiency of a deep reinforcement learner in Kulkarni et al. (2016).

On a different approach (Arie et al., 2012) also showed composing primitive actions through observation of a human teacher enables a robot to build sequences of actions in order to perform object manipulation tasks. This approach relies on neuroscience modeling of mirror neuron systems. From the computational neuroscience point of view for sequence-learning task with trial-and- error, Hikosaka et al. (1999) suggested that procedural learning proceeds as a gradual transition from a spatial sequence to a motor, based on observations that the brain uses two parallel learning processes to learn action sequences: spatial sequence (goal-oriented, task space) mechanism and motor sequence (policy space) mechanism. Each of the acquired motor sequences can also be used as an element of a more-complex sequence.

We would like to extend these ideas of representations of tasks as temporal abstraction and as hierarchies, and to exploit the dual representation of tasks and actions sequences in this paper. Instead of a pre-defined task hierarchy given by the programmer, our robot learner should be able to learn hierarchical representations of its task space to more easily use acquired skills for higher-level tasks.

### 1.3. Active Motor Learning in High-Dimensional Spaces

In order to learn sequences of primitive policies for multi-task learning, beyond the specific methods for learning sequences of policies and multi-task learning, we would like to review the methods for learning high-dimensional mappings. More specifically, while the cited works above have outlined the importance of the organization and order of the training data, we would like to examine how this organization can be decided online by the robot learner during its learning process, instead of being left to the designer or programmer.

To address the challenge of multi-task motor learning, we will take the point of view of continual learning, also named life-long or curriculum Bengio et al. (2009) learning, that constructs a sophisticated understanding of the world from its own experience to apply previously learned knowledge and skills to new situation with more complex skills and knowledge. Humans and other biological species have this ability to learn continuously from experience and use these as the foundation for later learning. Reinforcement learning, as described in Sutton and Barto (1998), has introduced in a framework for learning motor policies from experience by autonomous data sampling through exploration. However, classical techniques based on reinforcement learning such as Peters and Schaal (2008) and Stulp and Schaal (2011) still need an engineer to manually design a reward function for each particular task, limiting their capability for multi-task learning.

#### 1.3.1. Intrinsic Motivation

More recent algorithms have tried to replace this manually defined reward function, and have proposed algorithms using intrinsic reward, using inspiration from *intrinsic motivation*, which is first described in developmental psychology as triggering curiosity in human beings Deci and Ryan (1985), and has more recently been described in terms of neural mechanisms for information-seeking behaviors (Gottlieb et al., 2013). This theory tries to explain our ability to learn continuously, although we do not have a clear tangible goal other than survival and reproduction, intrinsically motivated agents are still able to learn a wide variety of tasks and specialize in some tasks influenced by their environment and development, even in some tasks that are not directly useful for survival and reproduction. Psychological theories such as intrinsic motivation have tried to explain these apparently non-rewarding behaviors and have been successfully inspired learning algorithms (Oudeyer et al., 2007; Schmidhuber, 2010). More recently, these algorithms have been applied for multi-task learning and have successfully driven the learner's exploration through goal-oriented exploration as illustrated in Baranes and Oudeyer (2010) and Rolf et al. (2010). Santucci et al. (2016) has also proposed a goal-discovering robotic architecture for intrinsically-motivated learning to discover goals and learn corresponding policies, providing the number of goals is preset. Intrinsic motivation has also been coupled with deep reinforcement learning in Colas et al. (2018) to solve sparse or deceptive reward problems to reach a single goal.

However for multi-task learning, especially when the dimension of the outcome space increases, these methods become less efficient (Baranes and Oudeyer, 2013) due to the curse of dimensionality, or when the reachable space of the robot is small compared to its environment. To enable robots to learn a wide range of tasks, and even an infinite number of tasks defined in a continuous space, heuristics such as social guidance can help by driving its exploration toward interesting and reachable space fast.

#### 1.3.2. Social Guidance

Indeed, imitation learning has proven very efficient for learning in high-dimensional space as demonstration can orient the learner toward efficient subspaces. Information could be provided to the robot using external reinforcement signals (Thomaz and Breazeal, 2008), actions (Grollman and Jenkins, 2010), advice operators (Argall et al., 2008), or disambiguation among actions (Chernova and Veloso, 2009). Furthermore, tutors' demonstrations can be combined with autonomous robot learning for more efficient exploration in the sensori-motor space. Initial human demonstrations have successfully initiated reinforcement learning in Muelling et al. (2010) and Reinhart (2017). Nguyen et al. (2011) has combined demonstrations with intrinsic motivation throughout the learning process and shown that autonomous exploration is bootstrapped by demonstrations, enabling the learner to learn mappings in higher-dimensional spaces. Another advantage of introducing imitation learning techniques is to include non-robotic experts in the learning process (Chernova and Veloso, 2009).

Furthermore, tutor's guidance has been shown to be more efficient if the learner can actively request a human for help when needed instead of being passive, both from the learner or the teacher perspective (Cakmak et al., 2010). This approach is called interactive learning and it enables a learner to benefit from both local exploration and learning from demonstration. One of the key elements of these hybrid approaches is to choose when to request human information or learn in autonomy so as to diminish the teacher's attendance.

#### 1.3.3. Strategic Learning

This principle of a learner deciding on its learning process is generalized as *strategic learning*, as formalized in Lopes and Oudeyer (2012). Simple versions have enabled the learner to choose which task space to focus on (Baranes and Oudeyer, 2010), or to to change its strategy online (Baram et al., 2004). In Nguyen and Oudeyer (2012), the algorithm SGIM-ACTS enabled the robot learner to both choose its strategy and target outcome. Owing to its ability to organize its learning process, by choosing actively both which strategy to use and which outcome to focus on,. They have introduced the notion of *strategy* as a method of generating actions and outcome samples This study considered 2 kinds of strategy: autonomous exploration driven by intrinsic motivation and imitation of one of the available human teachers. The SGIM-ACTS algorithm relies on the empirical evaluation of its learning progress. It showed its potential to learn on a real high dimensional robot a set of hierarchically organized tasks in Duminy et al. (2016). This is why we consider to extend SGIM-ACTS to learn to associate a large number of tasks to motor policy sequences.

However, these works have considered a policy space at fixed dimensionality, thus policies of bounded complexity. We would like to extend these methods for unbounded sequences of motor primitives and for larger outcome spaces.

### 1.4. Can Tutors' Demonstrations Help Learn Sequences of Policies and Task Hierarchies?

In this work in multi-task learning, we want to enable a robot learner to achieve a wide range of tasks that can be inter-related and complex. Based on the state of the art, we base our framework on a parallel representation of sequences of policies as temporal abstraction and sequences of outcomes, as well as a representation of task hierarchies. We will use heuristics of intrinsic motivation, social guidance and strategic learning to enable the robot to learn the high-dimensional mapping between sequences of primitive policies and outcomes, via the learning of the task hierarchies. Thus we will propose a framework for representing hierarchical relationships between tasks and propose a learning algorithm that enables the emergence of such a representation. We will examine the performance of our robot learner for all the tasks defined in the experimental setups according to the point of view of multi-task learning, and we will examine more precisely the performance of our robot learner in the most complex tasks to assess whether it was able to increase its skills. We allow the robot to use sequences of actions of undetermined length to achieve these tasks. The learning algorithm has to face the problem of unlearnability of infinite task and policy spaces, and the curse of dimensionality of sequences of high-dimensionality policy spaces. We developed in Duminy et al. (2018) a new framework called “procedures” (see section 2.2) which proposes to combine known policies represented in a goal-oriented way. This framework showed its ability to improve the learning process of autonomous learners in preliminary experiments.

In this article, we would like to confirm these results by statistical analysis, and most of all, show that interactive strategies can further bootstrap the learning process of such autonomous learners and to help the robot to learn a relevant representation of the hierarchies of tasks. In the next section, we detail our methods based on the procedures framework and the proposed learning algorithm. We will describe in section 3 an experiment, on which we have tested our algorithm, and we will present and analyze the results in section 4.

## 2. Our Approach

Inspired by developmental psychology, we combine interactive learning and autonomous exploration in a strategic learner, which learning process is driven by intrinsic motivation. This learner also takes task hierarchy into account to reuse its previously learned tasks while adapting the complexity of its policy sequence to the complexity of the task at hand.

In this section, we formalize our learning problem, introduce a goal-oriented representation of sequence of policies and explain the principles of the algorithm SGIM-PB, which is an extension of SGIM-ACTS for learning motor policy sequences of unlimited size. Then, combining it with this “procedures” framework, we developed a new algorithm called Socially Guided Intrinsic Motivation with Procedure Babbling (SGIM-PB) capable of determining a task hierarchy representation to learn a set of complex interrelated tasks using adapted policy sequences.

### 2.1. Problem Formalization

In our approach, an agent can perform motions through the use of primitive policies π_θ_, parameterized by $\theta \in P\subset {\mathbb{R}}^{n}$. It can also perform policy sequences, which are potentially unbounded sequences of primitive motor policies executed sequentially. The policy space $P^{\mathbb{N}}=\cup_{i\in \mathbb{N}}P^{i}$ is the combination of all subspaces $P^{i}$ corresponding to each number of primitives, and is a continuous space of infinite dimensionality. Those policies have an effect on the environment, which we call the outcome ω ∈ Ω. The agent is then to learn the mapping between the policy space $P^{\mathbb{N}}$ and Ω: it learns to predict the outcome ω of each policy π_θ_ (the forward model *M*), but more importantly, it learns which policy to choose for reaching any particular outcome (an inverse model *L*). The outcomes ω can be of composite nature and thus be split in subspaces Ω_*i*_ ⊂ Ω of different dimensionality. Policy sequences are represented by concatenating the parameters of each of its primitive policies in the execution order.

We take the trial and error approach, and suppose that Ω is a metric space, meaning the learner has a means of evaluating a distance between two outcomes *d*(ω_1_, ω_2_).

### 2.2. Procedures

As this algorithm tackles the learning of complex hierarchically organized tasks, exploring and exploiting this hierarchy could ease the learning of the more complex tasks. We define procedures as a way to encourage the robot to reuse previously learned tasks, and chain them to build more complex ones. More formally, a procedure is defined as a succession of previously known outcomes (ω_1_, ω_2_, …, ω_*n*_ ∈ Ω) and is noted (ω_1_, ω_1_, …, ω_*n*_). The procedure space is thus simply Ω^ℕ^. The definition of the procedure space only depends on the outcome space. But the valid procedures, representing the real dependencies between tasks, depend on each application case. Thus the learning agent can explore the procedure space to test which procedures are valid.

**Figure 1:**
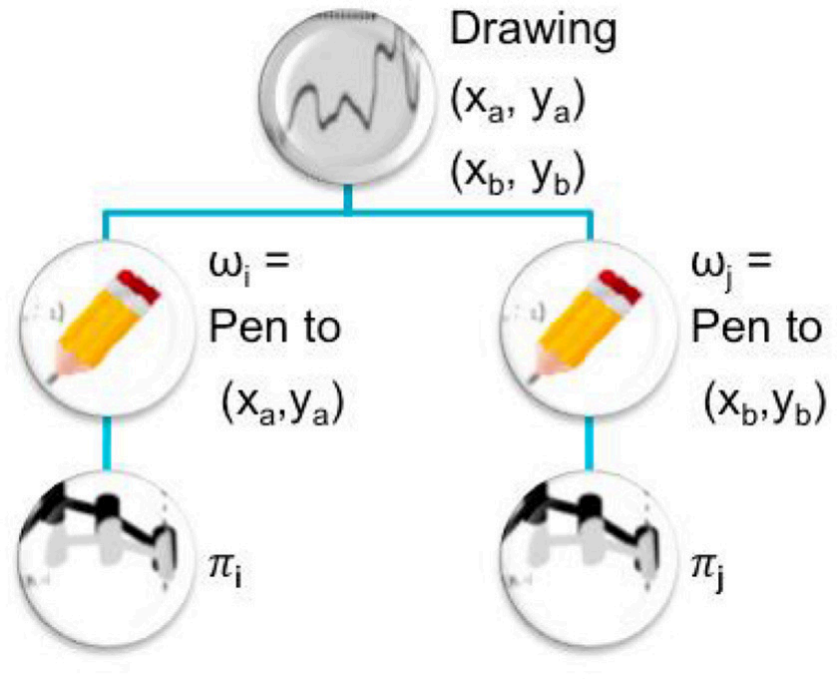
Illustration of a procedure or task hierarchy. To make a drawing between points (*x*_*a*_, *y*_*a*_) and (*x*_*b*_, *y*_*b*_), a robot can recruit subtasks consisting in (ω_*i*_) moving the pen to (*x*_*a*_, *y*_*a*_), then (ω_*j*_) moving the pen to (*x*_*b*_, *y*_*b*_). These subtasks will be completed respectively with policies π_*i*_ and π_*j*_. Therefore to complete the complete this drawing, the learning agent can use the sequence of actions (π_*i*_, π_*j*_).

Executing a procedure (ω_1_, ω_1_, …, ω_*n*_) means building the policy sequence π corresponding to the succession of policies π_*i*_, *i* ∈ ⟦1, *n*⟧ (potentially policy sequences as well) and execute it (where the π_*i*_ reach best the ω_*i*_ ∀*i* ∈ ⟦1, *n⟧* respectively). An example illustrates this idea of task hierarchy in Figure 1. As the subtasks ω_*i*_ are generally unknown from the learner, the procedure is updated before execution (see Algorithm 1) to subtasks $\omega_{i}^{\prime }$ which are the closest tasks reached by the learner (by executing respectively $\pi_{1}^{\prime }$ to $\pi_{n}^{\prime }$). When the agent selects a procedure to be executed, this latter is only a way to build the policy sequence which will actually be executed. So the agent does not check if the subtasks are actually reached when executing a procedure.

**Algorithm 1:** Procedure adaptation

**Input:** $\left(\omega_{1},\ldots ,\omega_{n}\right)\in \Omega^{n}$
**Input:** inverse model *L*
1: **for** *i* ∈ ⟦1, *n*⟧ **do**
2: $\omega_{i}^{\prime }\leftarrow$Nearest-Neighbor(ω_*i*_) // *get the nearest outcome known from* ω_*i*_
3: $\pi_{i}^{\prime }\leftarrow L\left(\omega_{i}^{\prime }\right)$ // *get the known policy sequence that reached* $\omega_{i}^{\prime }$
4: **end for**
5: **return** $\pi =\pi_{1}^{\prime }\ldots \pi_{n}^{\prime }$

If the procedure given can not be executed by the robot, because at least one of the subtasks space is not reachable, then the procedure is abandoned and replaced by a random policy sequence.

### 2.3. Socially Guided Intrinsic Motivation With Procedure Babbling

The SGIM-PB algorithm is the last achievement of a series of increasingly complex architectures that we have been developing for autonomous open-ended learning. It is an extension of SGIM-ACTS (Nguyen and Oudeyer, 2012), using the same interest model and memory based inverse model, but it can in addition perform sequences of motor policies. In this study, we limited our learning algorithm to the case of procedures of size 2 (sequences of 2 outcomes only) as we wish to prove the bootstrapping effect of the representation via procedures, before tackling the challenges of exploring a high-dimensional space of procedures Ω^ℕ^. This still allows the learning agent to use a high number of subtasks because of the recursivity of the definition of procedures. Our learning algorithm, called SGIM-PB, starts from scratch, it is only provided with the primitive policy space and outcome subspaces dimensionalities and boundaries. The procedural spaces are also predefined, as all the possible composition of outcome subspaces (Ω_*i*_, Ω_*j*_) with Ω_*i*_, Ω_*j*_ ⊂ Ω. Then its aim is to learn how to reach a set of outcomes as broad as possible, as fast as possible. This means it has to learn both the possible outcomes to reach and the policy sequences or procedures to use for that. In order to learn, the agent can count on different learning strategies, which are methods to build a policy or procedure from any given target outcome. It also need to map the outcome subspaces and even regions to the best suited strategies to learn them. In this algorithm, the forward and inverse models are memory based and consist only of the cumulative data, mappings of policies, procedures and their respective reached outcomes obtained through all the attempts of the learner. So they are learned by adding new data in the learner's memory.

**Figure 2:**
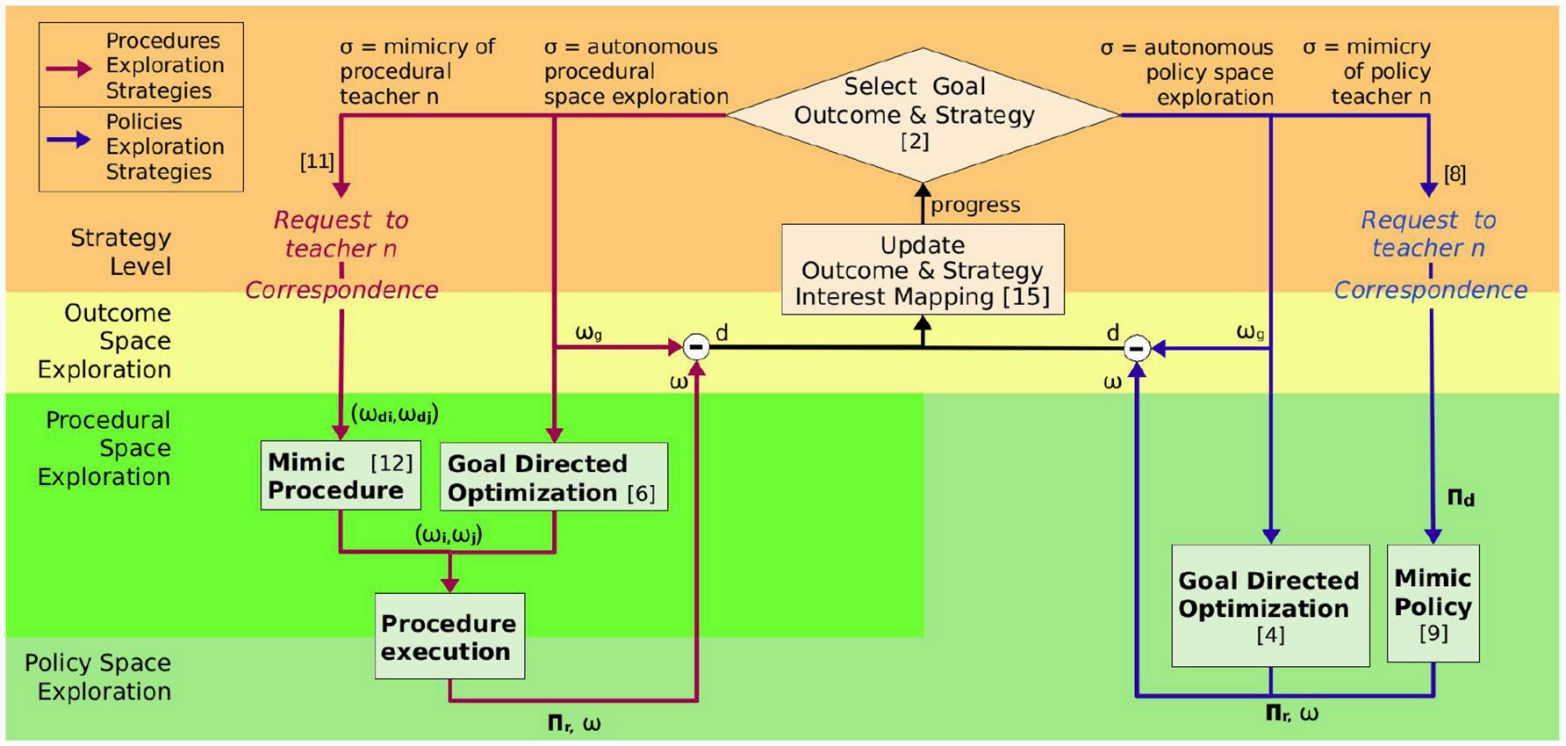
SGIM-PB architecture: number between brackets link parts of the architecture with corresponding lines in Algorithm 2, the arrows show the data transfer between the different blocks.

The SGIM-PB algorithm (see Algorithm 2, Figure 2) learns by episodes, where it starts by selecting an outcome ω_*g*_ ∈ Ω to target and an exploration strategy σ based on its progress as in most competence-based intrinsic motivation algorithms (Baranes and Oudeyer, 2010; Forestier and Oudeyer, 2016) and as detailed in section 2.3.2.

**Algorithm 2:** SGIM-PB

**Input:** the different strategies σ_1_, …, σ_*n*_
**Initialization:** partition of outcome spaces *R*←⊔_*i*_{Ω_*i*_}
**Initialization:** episodic memory *Memo* ←∅
1: **loop**
2: ω_*g*_, σ ← Select Goal Outcome and Strategy(*R*)
3: **if** σ = Autonomous policy space exploration strategy **then**
4: *Memo* ← Goal-Directed Policy Optimization(ω_*g*_)
5: **else if** σ = Autonomous procedural space exploration strategy **then**
6: *Memo* ← Goal-Directed Procedure Optimization(ω_*g*_)
7: **else if** σ = Mimicry of policy teacher *i* strategy **then**
8: (π_θ_*d*__, ω_*d*_)← ask and observe demonstrated policy to teacher *i*
9: *Memo* ← Mimic Policy(π_θ_*d*__)
10: **else if** σ = Mimicry of procedural teacher *i* strategy **then**
11: ((ω_*di*_, ω_*dj*_), ω_*d*_)← ask and observe demonstrated procedure to teacher *i*
12: *Memo* ← Mimic Procedure((ω_*di*_, ω_*dj*_))
13: **end if**
14: Update *L* with collected data *Memo*
15: *R* ← Update Outcome and Strategy Interest Mapping(*R*,*Memo*,ω_*g*_)
16: **end loop**

In each episode, the robot starts from the same position before executing a policy, and primitives are executed sequentially without getting back to this initial position. Whole policy sequences are recorded with their outcomes, but each step of the policy sequence execution is also recorded. These data enable the robot to select parts of the policy sequences, thus helping it to optimize the size of policy sequences it executes with respect to the outcomes at hand. The way these data are generated depend on the strategy chosen. We consider two *autonomous exploration* strategies (policy space exploration and procedural space exploration) and two which we call *socially guided exploration* (mimicry of a policy teacher and mimicry of a procedural teacher).

#### 2.3.1. Episodes Using Exploration Strategies

In an episode under the autonomous policy space exploration strategy (line 3 on Algorithm 2), the learner tries to optimize the policy π_θ_ to produce ω_*g*_ by choosing between random exploration of policies and local optimization, following the SAGG-RIAC algorithm (Baranes and Oudeyer, 2010) [Goal-Directed Policy Optimization (ω_*g*_)]. This choice is stochastic and based on the closeness of the goal outcome to already known outcomes, local optimization having a higher probability to be selected if the goal outcome neighborhood contains close known outcomes. Random exploration builds a random policy sequence recursively, starting by a random primitive policy, and adding more random primitives according to a probability of 1/α^*n*^, α = 2 being a constant and *n* the size of the already built policy sequence. Local optimization uses local linear regression. This is a slightly modified version of the SGIM-ACTS autonomous exploration strategy which interpolates from the known policies reaching an outcome close to ω_*g*_.

In an episode under the autonomous procedural space exploration strategy (line 5 on Algorithm 2), the learner builds a size 2 procedure (ω_*i*_, ω_*j*_) such as to reproduce the goal outcome ω_*g*_ the best using Goal-Directed Optimization [Goal-Directed Procedure Optimization(ω_*g*_)]. The procedure built is then modified and executed, following Algorithm 1.

In an episode under the mimicry of a policy teacher strategy (line 7 on Algorithm 2), the learner requests a demonstration π_θ_*d*__ from the chosen teacher. π_θ_*d*__ is selected by the teacher as the closest from the goal outcome ω_*g*_ in its demonstration repertoire. This repertoire is built in advance in practice for our experiments, by recording policies and their reached outcomes. The learner then repeats the demonstrated policy [Mimic Policy (π_θ_*d*__)]. It is a strategy directly also available in the SGIM-ACTS algorithm.

In an episode under the mimicry of a procedural teacher strategy (line 10 on Algorithm 2), the learner requests a procedural demonstration of size 2 (ω_*di*_, ω_*dj*_) which is built by the chosen teacher according to a preset function which depends on the target outcome ω_*g*_. Then the learner tries to reproduce the demonstrated procedure by refining and executing it, following Algorithm 1 [Mimic Procedure (ω_*di*_, ω_*dj*_)].

In both autonomous exploration strategies, the learner uses a method, Goal-Directed Optimization, to optimize its input parameters (procedure for the procedure exploration and policy for the policy exploration) to reach ω_*g*_ best. This generic method either creates random inputs, if the goal outcome ω_*g*_ is far from any previously reached one, or performs local optimization based on linear regression.

#### 2.3.2. Interest Mapping

After each episode, the learner stores the policies and modified procedures executed along with their reached outcomes in its episodic memory. It computes its *competence* in reaching the goal outcome ω_*g*_ by computing the distance *d*(ω_*r*_, ω_*g*_) with the outcome ω_*r*_ it actually reached. Then it updates its interest model by computing the interest *interest*(ω, σ) of the goal outcome and each outcome reached (including the outcome spaces reached but not targeted): *interest*(ω, σ) = *p*(ω)/*K*(σ), where *K*(σ) is the cost of the strategy used and the empirical progress *p*(ω) is the difference between the best competence before the attempt and the competence for the current attempt.

The learning agent then uses these interest measures to partition the outcome space Ω into regions of high and low interest. For each strategy σ, the outcomes reached and the goal are added to their partition region. Over a fixed number of measures of interest in the region, it is then partitioned into 2 subregions so as maximize the difference in interest between the 2 subregions. The method used is detailed in Nguyen and Oudeyer (2014). Thus, the learning agent discovers by itself how to organize its learning process and partition its task space into unreachable regions, easy regions and difficult regions, based on empirical measures of interest. This corresponds to line 15 on Algorithm 2.

The choice of strategy and goal outcome is based on the empirical progress measured in each region *R*_*n*_ of the outcome space Ω. This corresponds to the line 2 of Algorithm 2. ω_*g*_, σ are chosen stochastically (with respectively probabilities *p*_1_, *p*_2_, *p*_3_), by one of the sampling modes:

- mode 1: choose σ and ω_*g*_ ∈ Ω at random;
- mode 2: choose an outcome region *R*_*n*_ and a strategy σ with a probability proportional to its interest value. Then generate ω_*g*_ ∈ *R*_*n*_ at random;
- mode 3: choose σ and *R*_*n*_ like in mode 2, but generate a goal ω_*g*_ ∈ *R*_*n*_ close to the outcome with the highest measure of progress.

In the start of the learning process, as the robot has no outcome and interest measure to guide this choice, the first mode doing random exploration is automatically selected. At this state, the partition regions consist of the whole outcome subspaces.

The learner can compute nearest neighbors to select policies or procedures to optimize (when choosing local optimization in any of both autonomous exploration strategies and when refining procedures) or when computing the competence to reach a specific goal, it actually uses a performance metric (1) which also takes into account the complexity of the policy chosen:

(1) $$\begin{array}{r} perf\left(\omega_{g}\right)=d\left(\omega ,\omega_{g}\right)\gamma^{n} \end{array}$$

where d(ω, ω_*g*_) is the normalized Euclidean distance between the target outcome ω_*g*_ and the outcome ω reached by the policy, γ is a constant and *n* is equal to the size of the policy (the number of primitives chained).

### 2.4. Summary

To summarize, we have formalized in this section the problem of multi-task learning as the learning of an inverse model between a composite space of continuous set of outcomes and a space of policies of infinite dimension. The aim is to learn a mapping between outcomes (sometimes referred to as *tasks*) and policy sequences. The learning agent is provided with a set of predefined tasks via a space of outcomes it can observe and a metric to assess the performance of its trials. It can interact with the environment via primitive policies in a predefined space. We then introduced the framework of *procedures* as a goal-directed representation of sequences of primitive policies. To show that procedures can bootstrap the learning of policy sequences, we have proposed SGIM-PB as a learning algorithm that leverages several data collection *strategies* : goal-babbling for autonomous exploration, exploration of procedures, and social guidance to bootstrap the learning. SGIM-PB learns to reach an ensemble of outcomes, by mapping them to policies. As a means, we propose with SGIM-PB to take advantage of the dependencies between tasks. It explores the procedure space to learn these dependencies. Combining these procedures with the learning of simple policies to complete simple tasks, it can build sequences of policies to achieve complex tasks.

We expect the robot to organize its learning process, beginning by learning low-level tasks by exploring the policy space or by imitating the policy teachers. Once it has a good mastery of these low-level tasks, it can take advantage of the dependencies between tasks by exploring the procedural space or imitating the procedural teachers. It thus gradually improves its competence in high-level tasks.

The formalization and algorithmic architecture proposed can apply to multi-task motor learning problems in static environments. The requirements for an experimental setup are:

- to define the primitive policies of the robot in a finite dimensional space.
- to define the different outcomes the user is interested in. This requires (1) defining the variables from the sensors needed and a rough range of their values (we do not need a precise estimation as the algorithm is robust to overestimations of these ranges, see Nguyen and Oudeyer, 2014) (2) a measure for the robot to assess its own performance such as a distance, as in all intrinsic motivation based algorithms. This measure is used as an “internal reward” function. Contrarily to classical reinforcement learning problems, this reward function is not fine tuned to the specific goal at hand, but is a generic function for all the goals in the outcome space. We use a normalized Euclidean distance for all the outcomes in our experiments, in an attempt to show that the specification of tasks for our learning algorithm does not require a fine-tuning as with other reinforcement learning algorithms. We believe that our framework is also applicable to other distance measures. This definition of tasks is probably the most constraining condition, and does not yet scale up well to physical robots in the real world.
- the environment and robot can reset to an initial state, as in most reinforcement learning algorithms.

## 3. Experiment

In this study, we designed an experiment with a simulated robotic arm, which can move in its environment and interact with objects in it. We considered a setup with multiple tasks to learn, with tasks independent of each other and tasks that are interdependent. For interdependent tasks, we were inspired by tool use examples such as the setup proposed in Forestier et al. (2017). Our robot can learn an infinite number of tasks, grouped as 6 hierarchically organized types of tasks. The robot is capable of performing policy sequences of unrestricted size (i.e., consisting of any number of primitives), with primitive policies highly redundant and of high dimensionality.

### 3.1. Simulation Setup

**Figure 3:**
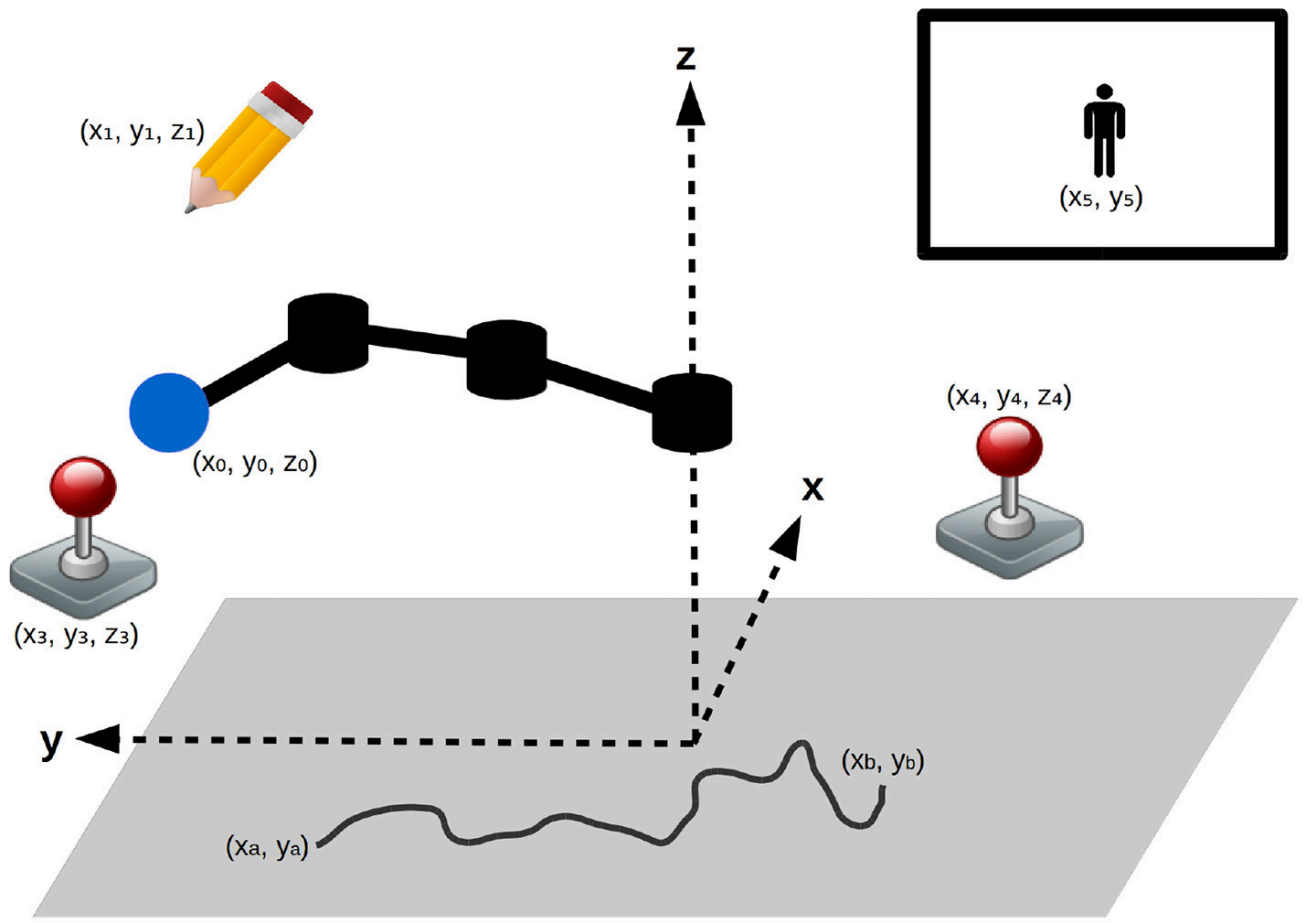
Experimental setup: a robotic arm, can interact with the different objects in its environment (a pen and two joysticks). Both joysticks enable to control a video-game character (represented in top-right corner). A gray floor limits its motions and can be drawn upon using the pen (a possible drawing is represented).

The Figure 3 shows environmental setup (contained in a cube delimited by (*x, y, z*) ∈ [−1; 1]^3^). The learning agent is a planar robotic arm of 3 joints with the base centered on the horizontal plane, able to rotate freely around the vertical axis (each link has a length of 0.33) and change its vertical position. The robot can grab objects in this environment, by hovering its arm tip (blue in the Figure 3) close to them, which position is noted (*x*_0_, *y*_0_, *z*_0_). The robot can interact with:

- Floor (below *z* = 0.0): limits the motions of the robot, slightly elastic which enable the robot to go down to *z* = −0.2 by forcing on it;
- Pen: can be moved around and draw on the floor, broken if forcing too much on the floor (when *z* <= −0.3);
- Joystick 1 (the left one on the figure): can be moved inside a cube-shaped area (automatically released otherwise, position normalized for this area), its *x*-axis position control a video-game character *x* position on the screen when grabbed by the robot;
- Joystick 2 (the right one on the figure): can be moved inside a cube-shaped area (automatically released otherwise, position normalized for this area), its *y*-axis position control a video-game character *y* position on the screen when grabbed by the robot;
- Video-game character: can be moved on the screen by using the two joysticks, its position is refreshed only at the end of a primitive policy execution for the manipulated joystick.

The robot grabber can only handle one object. When it touches a second object, it breaks, releasing both objects.

The robot always starts from the same position before executing a policy, and primitives are executed sequentially without getting back to this initial position. Whole policy sequences are recorded with their outcomes, but each step of the policy sequence execution is also recorded. This is done so as to enable the robot to select parts of policy sequences when it can, thus helping it to optimize the size of policy sequences it executes with respect to the outcomes at hand.

### 3.2. Experiment Variables

In this part, we formalize the parameters of the outcome space Ω and the policy space $P^{\mathbb{N}}$. The distance used to compare two policies together or two outcomes together is the normalized euclidean distance.

#### 3.2.1. Policy Spaces

The motions of each of the three joints of the robot are encoded using a one-dimensional Dynamic Movement Primitive (DMP) which are, as in Pastor et al. (2009), defined by the system:

(2) $$\begin{array}{rc} \tau \dot{v} & =K\left(g-x\right)-Dv+\left(g-x_{0}\right)f\left(s\right) \end{array}$$

(3) $$\begin{array}{rc} \tau ẋ & =v \end{array}$$

(4) $$\begin{array}{rc} \tau ṡ & =-\alpha s \end{array}$$

where *x* and *v* are the position and velocity of the system; *s* is the phase of the motion; *x*_0_ and *g* are the starting and end position of the motion; τ is a factor used to temporally scale the system (set to fix the length of a primitive execution); *K* and *D* are the spring constant and damping term fixed for the whole experiment; α is also a constant fixed for the experiment; and *f* is a non-linear term used to shape the trajectory called the forcing term. This forcing term is defined as:

(5) $$\begin{array}{r} f\left(s\right)=\frac{\sum_{i}w_{i}\psi_{i}\left(s\right)s}{\sum_{i}\psi_{i}\left(s\right)} \end{array}$$

where $\psi_{i}\left(s\right)=\exp\left(-h_{i}{\left(s-c_{i}\right)}^{2}\right)$ with centers *c*_*i*_ and widths *h*_*i*_ fixed for all primitives. There are 3 weights *w*_*i*_ per DMP.

The weights of the forcing term and the end positions are the only parameters of the DMP used by the robot. The starting position of a primitive is set by either the initial position of the robot (if it is starting a new policy sequence) or the end position of the preceding primitive. The robot can also set its position on the vertical axis *z* for every primitive. Therefore, a primitive policy π_θ_ is parameterized by:

(6) $$\begin{array}{r} \theta =\left(a_{0},a_{1},a_{2},z\right) \end{array}$$

where $a_{i}=\left(w_{0}^{\left(i\right)},w_{1}^{\left(i\right)},w_{2}^{\left(i\right)},g^{\left(i\right)}\right)$ corresponds to the DMP parameters of the joint *i*, ordered from base to tip, and *z* is the fixed vertical position. Thus, the primitive policies space is $P={\mathbb{R}}^{13}$. When combining two or more primitive policies (π_θ_0__, π_θ_1__, …), in a policy sequence π_θ_, the parameters (θ_0_, θ_1_, …) are simply concatenated together from the first primitive to the last. The total policy space, $P={\left({\mathbb{R}}^{13}\right)}^{\mathbb{N}}$ is of unbounded dimension.

#### 3.2.2. Outcome Subspaces

The outcome subspaces the robot learns to reach are hierarchically organized and defined as:

- Ω_0_: the position (*x*_0_, *y*_0_, *z*_0_) of the end effector of the robot in Cartesian coordinates at the end of a policy execution;
- Ω_1_: the position (*x*_1_, *y*_1_, *z*_1_) of the pen at the end of a policy execution if the pen is grabbed by the robot;
- Ω_2_: the first (*x*_*a*_, *y*_*a*_) and last (*x*_*b*_, *y*_*b*_) points of the last drawn continuous line on the floor if the pen is functional (*x*_*a*_, *y*_*a*_, *x*_*b*_, *y*_*b*_);
- Ω_3_: the position (*x*_3_, *y*_3_, *z*_3_) of the first joystick at the end of a policy execution if it is grabbed by the robot;
- Ω_4_: the position (*x*_4_, *y*_4_, *z*_4_) of the second joystick at the end of a policy execution if it is grabbed by the robot;
- Ω_5_: the position (*x*_5_, *y*_5_) of the video-game character at the end of a policy execution if moved.

The outcome space is a composite and continuous space $\Omega =\cup_{i=0}^{5}\Omega_{i}$, with subspaces of 3 to 4 dimensions. A quick analysis of this setup highlights interdependencies between tasks: controlling the position of the pen comes after controlling the position of the end effector; and controlling the position of the video-game character comes after controlling the positions of both joysticks, which in turn comes after controlling the position of the end effector. In our setup, the most complex task is controlling the position of the video-game character. This task should require a sequence of 4 actions : move the end-effector to initial position of the joystick 1, move joystick 1, then move the end-effector to the initial position of joystick 2, and move joystick 2. Besides, there are independent tasks: the position of the pen does not really depend on the position of the video-game character. Therefore, the inter-dependencies can be grouped into 2 dependency graphs. With this setup, we test if the procedures found by the robot can distinguish between dependent and independent tasks, and can compose tools uses.

The robot will choose at every episode a goal to reach in the outcome space Ω. In the beginning of its learning process, we expect the robot to make good progress in the easy tasks in Ω_0_ then Ω_1_, Ω_3_, Ω_4_ using *Autonomous policy space exploration* and *Mimicry of policy teacher* strategies. Once it has a good mastery of the easy tasks, it will concentrate on the more difficult tasks, and will benefit from procedures most, using *Autonomous procedural space exploration* and *Mimicry of procedural teacher* strategies.

In our multi-task learning perspective, we will examine how well the robot performs for each of the tasks in these subspaces. We will particularly examine its performance for the tasks of Ω_5_, which we consider the most complex tasks.

### 3.3. The Teachers

Our SGIM-PB learner can actively learn by asking teachers to give demonstrations of procedures or policies (strategies *Mimic procedural teacher* and *Mimic policy teacher*).

To help the SGIM-PB learner, procedural teachers were available so as to provide procedures for every complex outcome subspaces Ω_1_, Ω_2_, Ω_3_, Ω_4_ and Ω_5_. As Ω_0_ is the simplest outcome space in our setup, the base of its task hierarchy, we decided to build the preset functions for these procedural teachers up from Ω_0_. Each teacher was only giving procedures useful for its own outcome space, and was aware of its task representation. When presented with an outcome outside its outcome space of expertise, it provides a demonstration for a newly drawn random target outcome in its outcome space of expertise. They all had a cost of 5. The rules used to provide procedures are the following:

- ProceduralTeacher1 (ω_1_*g*__ ∈ Ω_1_): (ω_1_, ω_0_) with ω_1_ ∈ Ω_1_ equals to the pen initial position and ω_0_ ∈ Ω_0_ equals to the desired final pen position ω_1_*g*__;
- ProceduralTeacher2 (ω_2_*g*__ = (*x*_*a*_, *y*_*a*_, *x*_*b*_, *y*_*b*_) ∈ Ω_2_): (ω_1_, ω_0_) with ω_1_ ∈ Ω_1_ equals to the point on the *z* = 1.0 plane above the first point of the desired drawing ω_1_ = (*x*_*a*_, *y*_*a*_, 1), and ω_0_ ∈ Ω_0_ equals to the desired final drawing point, ω_0_ = (*x*_*b*_, *y*_*b*_, 0);
- ProceduralTeacher3 (ω_3_*g*__ ∈ Ω_3_): (ω_3_, ω_0_) with ω_3_ = (0, 0, 0), ω_3_ ∈ Ω_3_ and ω_0_ ∈ Ω_0_ equals to the end effector position leading to the desired final position of the first joystick ω_3_*g*__;
- ProceduralTeacher4 (ω_4_*g*__ ∈ Ω_4_): (ω_4_, ω_0_) with ω_4_ = (0, 0, 0), ω_4_ ∈ Ω_4_ and ω_0_ ∈ Ω_0_ equals to the end effector position leading to the desired final position of the second joystick ω_4_*g*__;
- ProceduralTeacher5 (ω_5_*g*__ = (*x, y*) ∈ Ω_5_): (ω_3_, ω_4_) with ω_3_ = (*x*, 0, 0), ω_3_ ∈ Ω_3_ with *x* corresponding to the desired x-position of the video-game character, ω_4_ = (0, *y*, 0), ω_4_ ∈ Ω_4_ with *y* corresponding to the desired y-position of the video-game character.

We also added policy teachers corresponding to the same outcome spaces to bootstrap the robot early learning process. The strategy attached to each teacher has a cost of 10. Each teacher was capable to provide demonstrations (as policies executable by the robot) linearly distributed in its outcome space. All those teachers consist of demonstrations repertoires built by drawing sparse demonstrations from a random policy learner trained a huge amount of time (1,000,000 iterations):

- MimicryTeacher1 (Ω_1_): 15 demonstrations;
- MimicryTeacher2 (Ω_2_): 25 demonstrations;
- MimicryTeacher3 (Ω_3_): 18 demonstrations;
- MimicryTeacher4 (Ω_4_): 18 demonstrations;
- MimicryTeacher5 (Ω_5_): 9 demonstrations;

These costs were chosen so as to encourage the robot to rely on itself as much as possible to reduce the teacher load. The costs of 10 for a policy teacher strategy and 5 for a procedural teacher are arbitrary. Their difference comes from our belief that giving a procedure takes less time to the teacher than providing it with a detailed demonstrated motor policy.

### 3.4. Evaluation Method

To evaluate our algorithm, we created a benchmark dataset for each outcome space Ω_*i*_, linearly distributed across the outcome space dimensions, for a total of 27,600 points. The evaluation consists in computing the normalized Euclidean distance between each of the benchmark outcome and their nearest neighbor in the learner dataset. Then we compute the mean distance to benchmark for each outcome space. The global evaluation is the mean evaluation for the 6 outcome spaces. This evaluation is repeated across the learning process at predefined and regularly distributed timestamps.

Then to asses our algorithm efficiency, we compare its results with 3 other algorithms:

- SAGG-RIAC: performs autonomous exploration of the policy space $P^{\mathbb{N}}$ guided by intrinsic motivation;
- SGIM-ACTS: interactive learner driven by intrinsic motivation. Choosing between autonomous exploration of the policy space $P^{\mathbb{N}}$ and mimicry of one of the available policy teachers;
- IM-PB: performs both autonomous exploration of the procedural space and the policy space, guided by intrinsic motivation;
- SGIM-PB: interactive learner driven by intrinsic motivation. Choosing between autonomous exploration strategies (either of the policy space or the procedural space) and mimicry of one of the available teachers (either policy or procedural teachers).

For each run for all algorithms, we let the algorithm perform arbitrarily 25,000 iterations (policy sequences executions or learning episodes). The value of γ for this experiment is 1.2. The probabilities to choose either of the sampling mode of SGIM-PB are *p*_1_ = 0.15, *p*_2_ = 0.65, *p*_3_ = 0.2. The code run for this experiment can be found in https://bitbucket.org/smartan117/sgim_iclr.

## 4. Results

### 4.1. Distance to Goals

**Figure 4:**
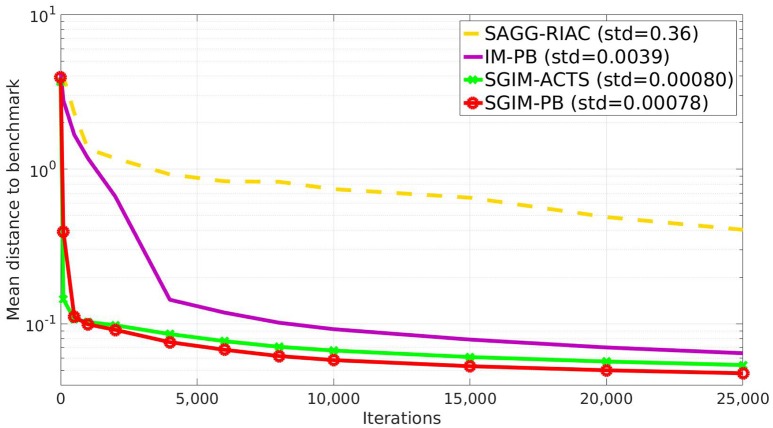
Evaluation of all algorithms (final standard deviation shown in caption).

The Figure 4 shows the global evaluation of all the tested algorithms, which corresponds to the mean error made by each algorithm to reproduce the benchmarks with respect to the number of complete policy sequences tried. Random, SGIM-ACTS, SGIM-PB were run 20 times while IM-PB and SAGG-RIAC was run 10 times on this setup so as to obtain statistically significant differences between SGIM-PB and the other algorithms, according to the Student's *t*-test on two algorithms : *p* = 3 ∗ 10^−16^ < 0.1 when compared with random, *p* = 0.01 for SAGG-RIAC, *p* = 1 ∗ 10^−9^ for SGIM-ACTS. The complete results for Student's *t*-test are reported in Table A1 in the Annex. The algorithms capable of performing procedures (IM-PB and SGIM-PB) have errors that drop to levels lower than the their non-procedure equivalents (SAGG-RIAC and SGIM-ACTS). The *t*-test comparing the final errors of IM-PB and SGIM-PB vs. SAGG-RIAC and SGIM-ACTS gives a strong difference with *p* = 9*e*−4 < 0.1. Moreover, this difference starts since the beginning of the learning process (shown on Figure 4). It seems that the procedures bootstrap the exploration, enabling the learner to progress further. Indeed, the autonomous learner IM-PB learner, the upgraded version of SAGG-RIAC by the use of procedures, has significantly better performance.

We can also see that the SGIM-PB algorithm has a very quick improvement in global evaluation owing to the bootstrapping effect of the different teachers. It goes lower to the final evaluation of SAGG-RIAC (0.17) after only 500 iterations. This bootstrapping effect comes from the mimicry teachers, as it is also observed for SGIM-ACTS which shares the same mimicry teachers.

**Figure 5:**
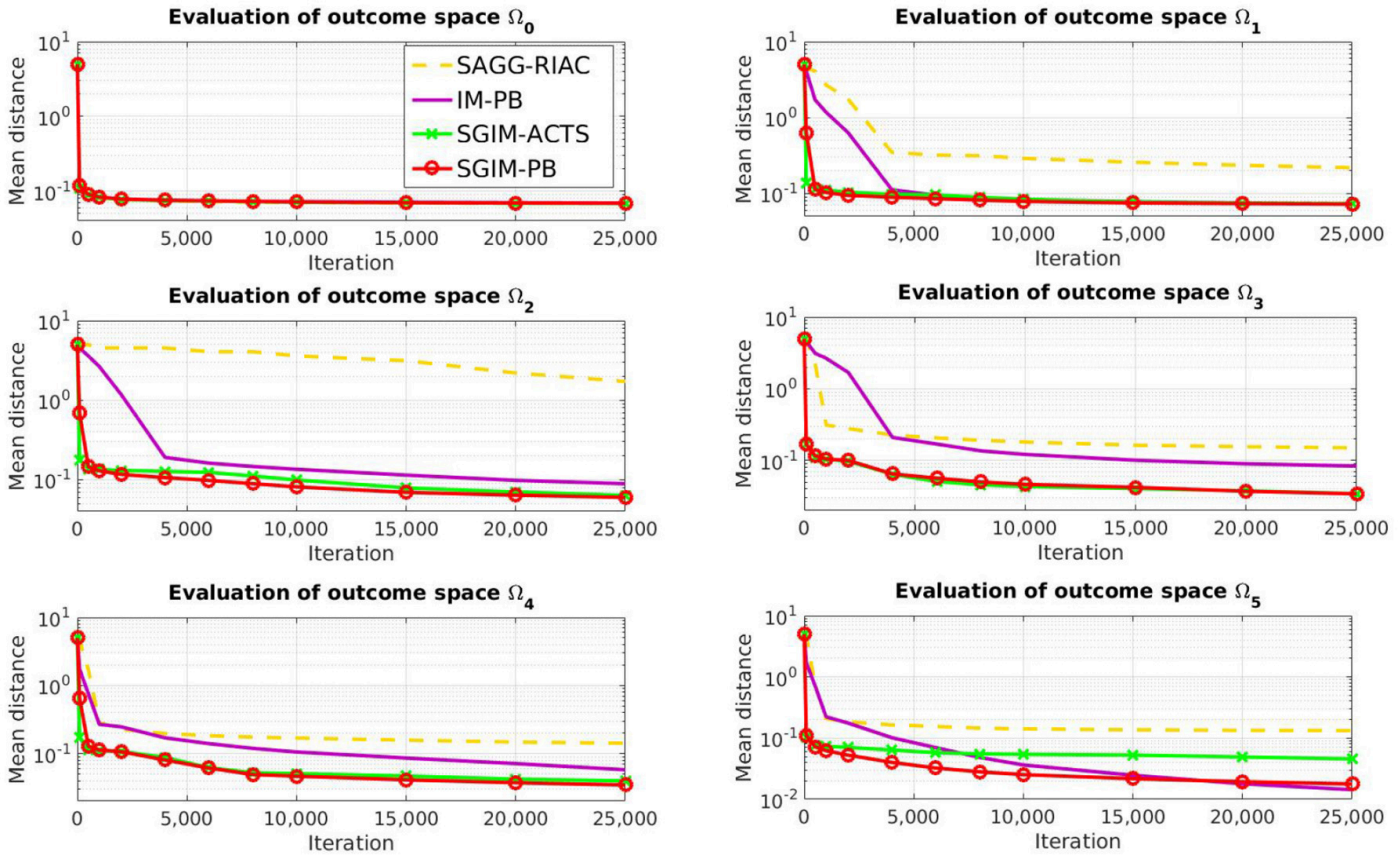
Evaluation of all algorithms per outcome space (for Ω_0_, all evaluations are superposed).

If we look at the evaluation on each individual outcome space (Figure 5), we can see that the learners with demonstrations (SGIM-PB and SGIM-ACTS) outperform the other algorithms, except for the most simple outcome space Ω_0_, which does not require sequences of actions, and the outcome space Ω_5_. In the case of Ω_5_, the difference with IM-PB is not significative (IM-PB seems a bit better but the difference is not significative with *p* > 0.1). The results for Student's *t*-test are reported in Table A1 in the Annex. This exception for Ω_5_ is due to the fact that IM-PB practiced much more on this outcome space (1500 iterations where it chose goals in Ω_5_ against 160 for SGIM-PB). SGIM-PB and SGIM-ACTS are much better than the other algorithms on the two joysticks outcome spaces (Ω_3_ and Ω_4_) (with respectively p=7e-4 and 1e-5). This is not surprising given the fact that those outcome spaces require precise policies. Indeed, if the end-effector gets out of the area where it can control the joystick, the latter is released, thus potentially ruining the attempt. So on these outcome spaces working directly on carefully crafted policies can alleviate this problem, while using procedures might be tricky, as the outcomes used don't take into account the motion trajectory but merely its final state. SGIM-PB was provided with such policies by the policy teachers. Also if we compare the results of the autonomous learner without procedures (SAGG-RIAC) with the one with procedures (IM-PB), we can see that it learns less on any outcome space but Ω_0_ (which was the only outcome space reachable using only single primitive policies and that could not benefit from using the task hierarchy to be learned) and especially for Ω_1_, Ω_2_ and Ω_5_ which were the most hierarchical in this setup. More generally, it seems than on this highly hierarchical Ω_5_, the learners with procedures were better. So the procedures helped when learning any potentially hierarchical task in this experiment.

### 4.2. Analysis of the Sampling Strategy Chosen for Each Goal

**Figure 6:**
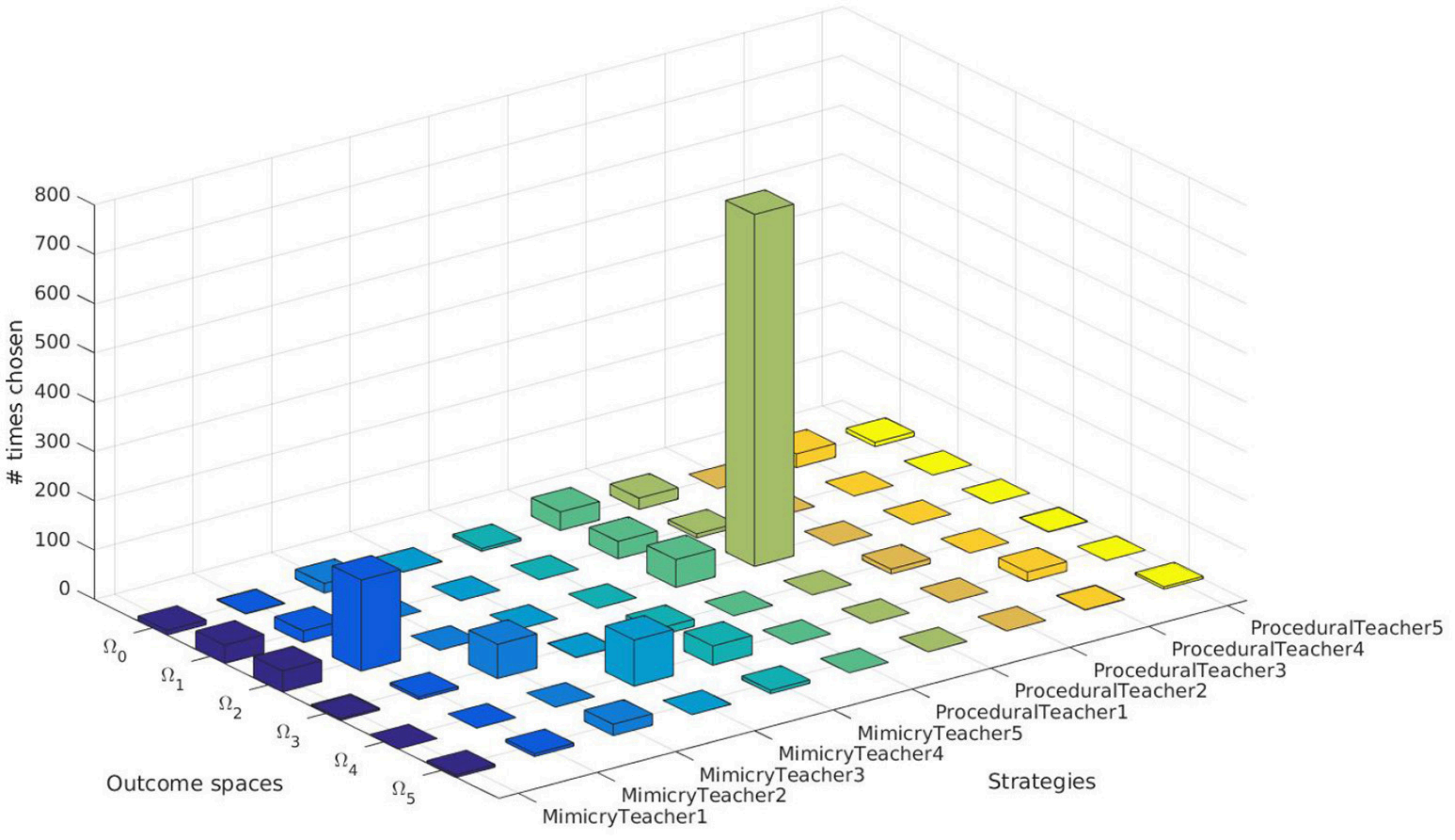
Choices of teachers and target outcomes of the SGIM-PB learner.

We further analyzed the results of our SGIM-PB learner. We looked in its learning process to see which pairs of teachers and target outcomes it has chosen (Figure 6). It was capable to request demonstrations from the relevant teachers depending on the task at hand, except for the outcome space Ω_0_ which had no human teachers and therefore could not find a better teacher to help it. Indeed, for the outcome space Ω_2_, the procedural teacher (ProceduralTeacher2) specially built for this outcome space was greatly chosen.

**Figure 7:**
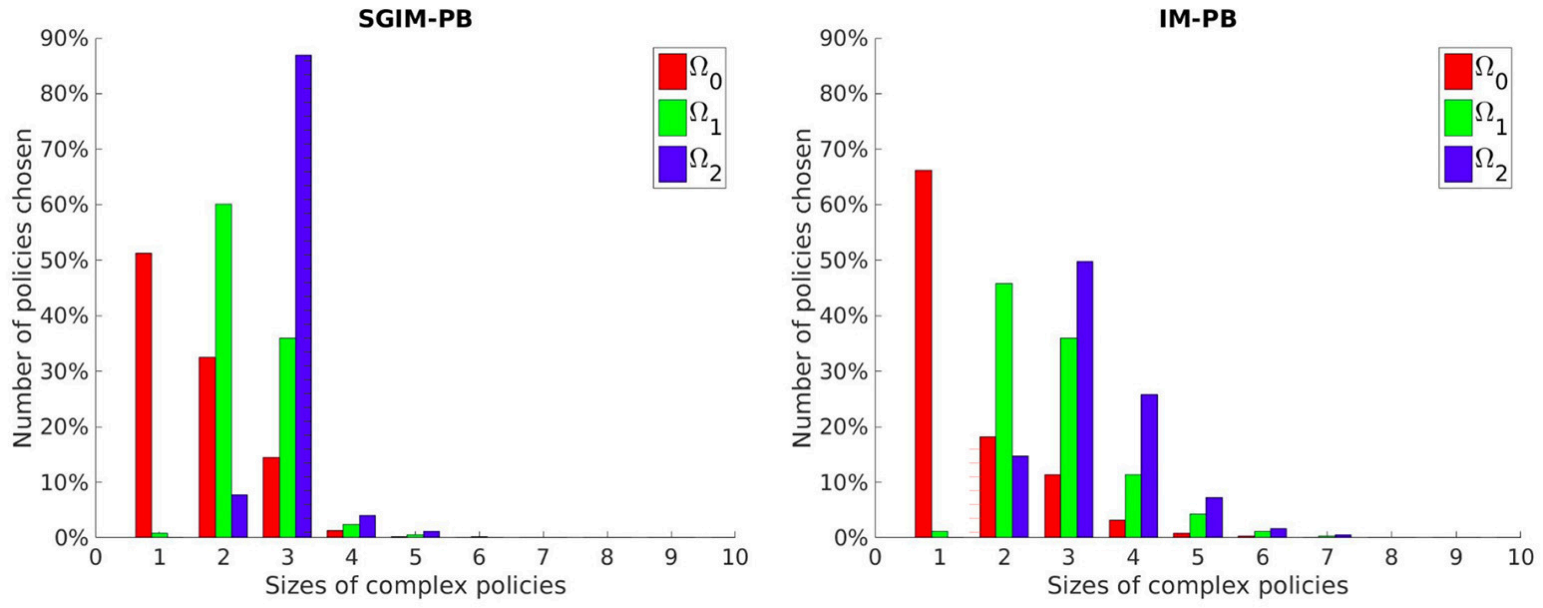
Number of policies selected per policy size for three increasingly more complex outcome spaces by the SGIM-PB (on the left) and IM-PB (on the right) learners.

We wanted to see if our SGIM-PB learner adapts the complexity of its policies to the working task. So we looked which policy space would be chosen by the local optimization function (used inside the policy space exploration strategy) for the Ω_0_, Ω_1_ and Ω_2_ subspaces (chosen because they are increasingly complex) on their respective evaluation benchmarks. We compared those results with the same obtained by the IM-PB learner to see if the teachers had an effect on the complexity of policies produced. Figure 7 shows the results of this analysis.

### 4.3. Length of the Sequence of Primitive Policies

As we can see on those three interrelated outcome subspaces (Figure 7), the learner is capable to adapt the complexity of its policies to the outcome at hand. It chooses longer policies for the Ω_1_ subspace (policies of size 2 and 3 while using mostly policies of size 1 and 2 for Ω_0_) and even longer for the Ω_2_ subspace (using far more policies of size 3 than for the others). It shows that our learner is capable to correctly limit the complexity of its policies instead of being stuck into always trying longer and longer policies. Also, if we look at the policy complexity of the IM-PB learner, we see it was also capable to correctly limit its complexity (especially on Ω_0_ where it used even more single-primitive policies than SGIM-PB). However, we can see that our SGIM-PB learner, owing to the teacher strategies available to it, had a smaller spread on the size of policy sequences distribution for each of the three outcome spaces.

We also wanted to see if our SGIM-PB algorithm had discovered the task hierarchy of this experiment. We hoped it would correctly assess which procedural space is adapted to each of the complex outcome subspaces (all subspaces except Ω_0_ as it cannot benefit from procedures to be reached). So we looked which procedural space was selected by the local optimization function (used inside the procedural space exploration strategy) for each of the outcome subspaces on their respective evaluation benchmarks. For assessing those results, we compared them with those obtained by the IM-PB learner on the same process.

**Figure 8:**
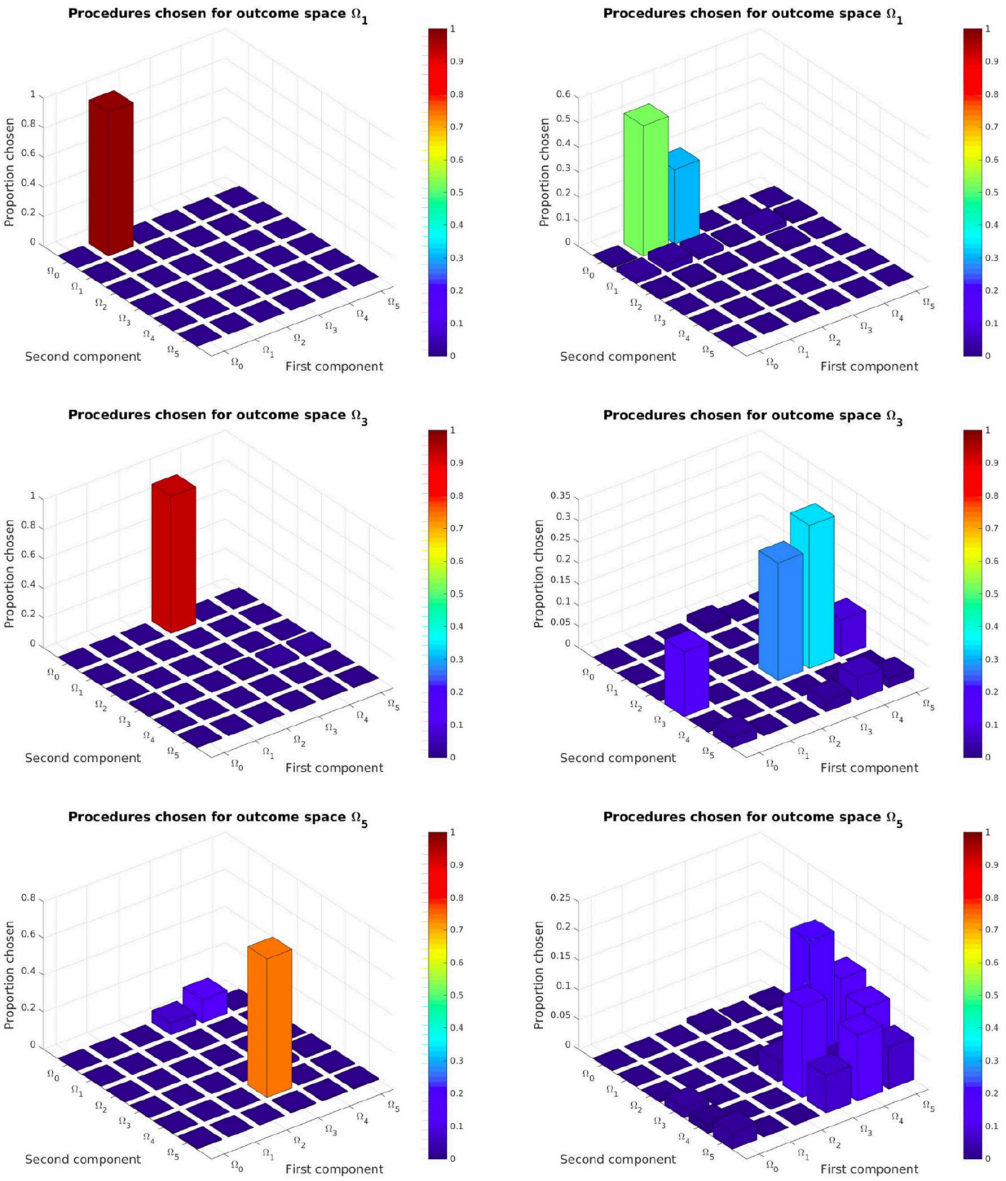
Task hierarchy discovered by the SGIM-PB (left side) and IM-PB (right side) learners for the outcome spaces Ω_1_, Ω_3_, Ω_5_: this represents for each complex outcome space the percentage of time each procedural space would be chosen. See Appendix A for the complete figure on Figure A1.

**Figure A1:**
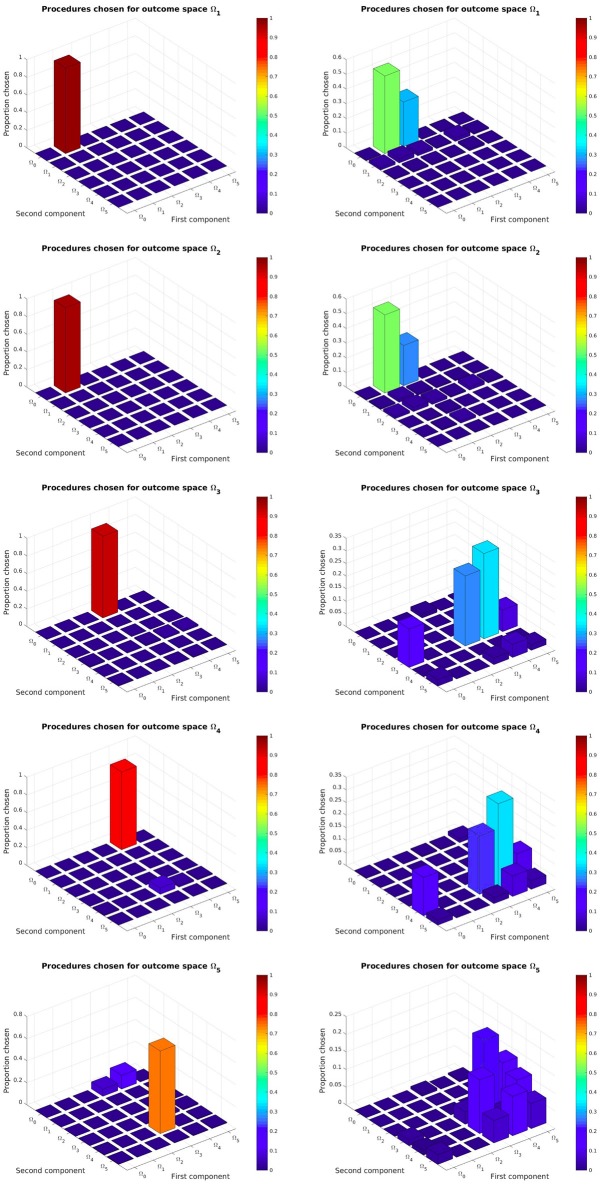
Task hierarchy discovered by the SGIM-PB (left side) and IM-PB (right side) learners: this represents for each complex outcome space the percentage of time each procedural space would be chosen.

As we can see on left column of Figure 8 and Figure A1, our SGIM-PB learner successfully chooses the procedural spaces most adapted for each complex outcome subspace (the same as those we used to build the procedural teachers). For instance, to move the video character (task Ω_5_), the robot mainly uses subtasks Ω_4_ (position of the second joystick) and Ω_3_ (position of the first joystick). To move the position of the first joystick (task Ω_3_), subtasks Ω_0_ (position of the end-effector) and Ω_3_ (position of the first joystick) are used. The same way, task Ω_4_ recruits subtasks Ω_0_ and Ω_4_. Thus by recursively, the robot has built a hierarchical representation that task Ω_5_ depends on subtasks (Ω_0_, Ω_4_, Ω_0_, Ω_3_). This means it was successfully able to discover and exploit it. By comparison, the IM-PB learner was only capable to identify useful procedural spaces for the Ω_1_ and Ω_2_ outcome subspaces. For both those outcome subspaces, it identified the one procedural space mainly used by SGIM-PB learner and another one (Ω_2_, Ω_0_) which can also be useful to learn to reach those outcome subspaces, though arguably less efficient. Indeed, using a policy moving the pen (in Ω_1_) is enough for the first component of procedures used to reach Ω_1_ and Ω_2_, and it can lead to less complex policy sequences than using one drawing on the floor (in Ω_2_). If we look at the result for the outcome subspaces Ω_3_ and Ω_4_, the IM-PB learner was incapable to identify adapted procedural spaces. The absence of a policy teacher to guide it could explain the IM-PB learner poor results on those outcome subspaces. Also, compared to the great focus of the SGIM-PB learner on this outcome subspaces, IM-PB results were more dispersed, indicating its difficulty to select an adapted procedural space. As those outcome subspaces require precise policies and are less adapted to procedures, this difficulty is understandable. By looking at the results of both learners, we can see that the procedural teachers had a profound impact on the choice of adapted procedures for each outcome subspaces, and clearly guided its whole learning process by helping it discover the task hierarchy of the experimental setup.

## 5. Conclusion and Future work

### 5.1. Conclusion

With this experiment, we show the capability of SGIM-PB to tackle the learning of a set of multiple interrelated complex tasks. It successfully discovers the hierarchy between tasks and uses sequences of motor policies to learn a wider range of tasks. It is capable to correctly choose the most adapted teachers to the target outcome when available. Though it is not limited in the size of policies it could execute, the learner shows it could adapt the complexity of its policies to the task at hand.

The procedures greatly improved the learning capability of autonomous learners, as shows the difference between IM-PB and SAGG-RIAC. Our SGIM-PB shows it is capable to use procedures to discover the task hierarchy and exploit the inverse model of previously learned tasks. More importantly, it shows it can successfully combine the ability of SGIM-ACTS to progress quickly in the beginning (owing to the mimicry teachers) and the ability of IM-PB to progress further on highly hierarchical tasks (owing to the procedure framework).

### 5.2. Contributions

In this article, we aimed to enable a robot to learn sequences of actions of undetermined length to achieve a field of outcomes. To tackle this high-dimensionality learning between a continuous high-dimensional space of outcomes and a continuous infinite dimensionality space of sequences of actions, we used techniques that have proven efficient in previous studies: goal-babbling, social guidance and strategic learning based on intrinsic motivation. We extended them with the procedures framework and proposed SGIM-PB algorithm, allowing the robot to babble in the procedure space and to imitate procedural teachers. We showed that SGIM-PB can discover the hierarchy between tasks, learn to reach complex tasks while adapting the complexity of the policy. Although the representation of actions and tasks are predefined, we described a developmental process involved in the emergence of representations of tasks highlighting their relationships. The study shows that:

- procedures allow the learner to learn complex tasks, and adapt the length of sequences of actions to the complexity of the task
- social guidance bootstraps the learning owing to demonstrations of primitive policy in the beginning, and then to demonstrations of procedures to learn how to compose tasks into sequences of actions
- intrinsic motivation can be used as a common criteria for active learning for the robot to choose both its exploration strategy, its goal outcomes and the goal-oriented procedures.

Our contributions in the field of cognitive robotics are to highlight (1) the relevance of a parallel representation of sequences in the action and the task space, through a goal-oriented temporal abstraction (2) the importance of a hierarchical representation of tasks in multi-task learning problems, and (3) the efficiency of active strategical learning in curriculum learning. An intrinsically motivated robot can learn how to collect data in an organized and meaningful order, from simple to more complex tasks. We have presented a developmental process involved in the emergence of representations of action and tasks.

### 5.3. Future Work

However a precise analysis of the impact of each of the different strategies used by our learning algorithm could give us more insight in the roles of the teachers and procedures framework. Also, we aim to illustrate the potency of our SGIM-PB learner on a real-world application. We are currently designing such an experiment with a physical robotic platform.

Besides, the procedures are defined as combinations of any number of subtasks but the algorithm we submitted only uses procedures as combinations of 2 subtasks. Because of the recursive definition of procedures, the robot can still have representations of complex tasks as composed of numerous subtasks. However, in order to have a direct representation of an unbounded number of tasks, it could be a next step to see if the learning algorithm can handle the curse of dimensionality of a larger procedure space, and explore combinations of any number of subtasks. Moreover, the algorithm can be extended to allow the robot learner to decide on how to execute a procedure. In the current version, we have proposed the “refinement process” to infer the best policy. We could make this refinement process more recursive, by allowing the algorithm to select, not only policies, but also lower-level procedures as one of the policy components.

## Author Contributions

ND wrote most parts of the paper. SN wrote more specifically parts of the introduction and of the approach parts. She also proposed corrections to the article, as did DD, who also oriented the research as ND thesis director.

### Conflict of Interest Statement

The authors declare that the research was conducted in the absence of any commercial or financial relationships that could be construed as a potential conflict of interest.

## Appendix

**Table A1:** Student's *t*-test on two samples for comparing SGIM-PB with each of the algorithms and for comparing the procedure algorithms (SGIM-PB and IM-PB) to algorithms without the procedure framework (SGIM-ACTS, SAGG-RIAC and random).

		**Global**	**Task0**	**Task 1**	**Task 2**	**Task 3**	**Task 4**	**Task 5**
SGIM-PB vs. random	t	–33	9	–27	–15	–32	–50	–57
	p	3e-16	5e-8	9e-15	4e-11	4e-16	6e-19	5e-20
SGIM-PB vs. SAGG-RIAC	t	–3	9	–10	–2	–44	–46	–84
	p	1e-2	6e-8	1e-8	3e-2	4e-18	2e-18	1e-22
SGIM-PB vs. IM-PB	t	–11	–4	–4	–5	–5	–3	1
	p	3e-9	4e-4	1e-3	1e-4	9e-5	3e-3	0.2
SGIM-PB vs. SGIM-ACTS	t	–12	5	–3	–3	–0.5	–3	–18
	p	1e-9	2e-4	2e-3	1e-2	6e-2	1e-2	2e-12
(SGIM-PB, IM-PB) vs.	t	–2.5	9	–5	–2	–4	–5	–8
(random, SAGG-RIAC, SGIM-ACTS)	p	2e-2	1e-12	3e-6	7e-2	6e-4	3e-6	4e-11

*We tested the difference of the distances to goal at the end of the learning (t = 25,000) for the global evaluation and for each task type. Negative values for t mean that SGIM-PB makes lower error. The non-significative results (p > 0.1) are highlighted*.
